# A Unique Case of Atypical Hemolytic Uremic Syndrome Secondary to Alcohol-Induced Acute Pancreatitis

**DOI:** 10.7759/cureus.109822

**Published:** 2026-05-28

**Authors:** Tara C Prakash, Yashaswi Koguru, Chirag Lodha

**Affiliations:** 1 Cell Biology and Physiology, Edward Via College of Osteopathic Medicine, Spartanburg, USA; 2 Internal Medicine, Grand Strand Medical Center, Myrtle Beach, USA; 3 Internal Medicine, University of South Florida (USF) Health, Tampa, USA

**Keywords:** alcoholic pancreatitis, atypical hemolytic syndrome, hematology, hemolytic uremic syndrome, microangiopathy, oncology

## Abstract

Atypical hemolytic uremic syndrome (aHUS), also known as complement-mediated thrombotic microangiopathy (CM-TMA), is an extremely rare but potentially life-threatening disorder that presents with the triad of thrombocytopenia, hemolytic anemia, and acute renal failure. aHUS is attributed to genetic mutations in the complement regulation system and is typically triggered by events such as pregnancy, vaccination, or surgery. This case report describes a patient initially admitted for acute pancreatitis secondary to alcohol abuse who developed aHUS within 48 hours of admission. The diagnosis of aHUS was confirmed via a full diagnostic workup. Hematology recommended supportive care. The case was further complicated by acute hypoxic respiratory failure secondary to community-acquired pneumonia, which developed simultaneously with the aHUS. The patient’s aHUS resolved as the acute pancreatitis resolved with supportive care, and the pneumonia resolved with antibiotics and inhaled corticosteroids, which likely helped improve the aHUS. When patients present with acute pancreatitis and hematologic abnormalities, clinicians should suspect aHUS and perform a thorough workup to distinguish between aHUS and other autoimmune hematologic conditions, such as typical hemolytic uremic syndrome (HUS), thrombotic thrombocytopenic purpura (TTP), or drug-induced complement-mediated anemia (CMA). While most reported cases have been treated with plasma exchange therapy or eculizumab, we present one of the few cases where the patient was treated with supportive care.

## Introduction

Atypical hemolytic uremic syndrome (aHUS) is a thrombotic microangiopathy (TMA) that presents with the triad of thrombocytopenia, hemolytic anemia, and acute renal failure. “Atypical” is used to differentiate the condition from typical hemolytic uremic syndrome (HUS), which is associated with diarrhea or Shiga toxin-producing *Escherichia coli *[1].

aHUS may be unprovoked, which is known as primary aHUS, or it may develop secondary to an external trigger, in which case it is referred to as secondary aHUS [2]. Primary aHUS is often associated with genetic mutations affecting complement regulatory pathways, including variants in membrane cofactor protein (MCP), complement factor H (CFH), and complement component 3 (C3). It is thought that these mutations result in dysregulated complement activation, ultimately leading to endothelial injury and organ damage [1]. In contrast, these genetic abnormalities are typically absent in secondary aHUS. In secondary aHUS, a precipitating event, most commonly drugs, autoimmune disease, or infection, promotes endothelial injury and the release of proinflammatory and procoagulant cytokines, leading to complement activation and subsequent disease development [2]. Although acute pancreatitis has been identified in the literature as a potential trigger for aHUS, the relationship is exceedingly rare, with fewer than 40 cases reported since 1978 [3]. Notably, alcohol-induced pancreatitis is the most common type of pancreatitis that triggers aHUS [4].

Both primary and secondary aHUS are rare, with an estimated prevalence of one in 500,000 [5]. However, when aHUS does occur, early diagnosis is critical as aHUS can quickly progress to a life-threatening illness. About 40% of patients with aHUS require dialysis at diagnosis [4], and aHUS itself carries a mortality rate as high as 25% [3]. Furthermore, the literature suggests that management of secondary aHUS should primarily focus on identifying and treating the underlying cause, given the nature of the pathophysiology of secondary aHUS [2]. Despite this, in clinical practice, secondary aHUS is still typically treated with a complement inhibitor or plasma exchange therapy, with only a small number of reported cases focusing treatment on the underlying cause [4-6].

In this report, we discuss a case of aHUS secondary to alcohol-induced pancreatitis that resolved with supportive treatment. The extremely rare occurrence of aHUS in the setting of alcohol-induced acute pancreatitis highlights the clinical significance of this case, as well as the importance of having TMA on the differential in patients with pancreatitis. Furthermore, the case was managed with supportive care alone, without the need for complement inhibition therapy. This clinical course further supports addressing the underlying condition in cases of secondary aHUS.

## Case presentation

A 43-year-old Caucasian male patient with a past medical history of gastroesophageal reflux disease (GERD), hypertension, and alcohol use disorder (AUD) presented to the emergency department with intense generalized abdominal tenderness, which began a few hours prior to arrival. He complained of nausea but denied any vomiting. He reported that he had similar episodes every few months but had not had any recent hospitalizations. He denied any recent imaging of the abdomen. His surgical history consisted of a cholecystectomy performed at an unknown date. He denied any blood in the stool or urine, as well as any problems urinating or defecating. The patient reported drinking about 18 beers every weekend. However, there was no history of alcohol withdrawal or documented history of cirrhosis. He denied any illicit drug use.

On presentation, blood pressure was elevated at 167/96 mmHg, and the rest of his vitals were within normal limits. Physical exam was positive for moderate diffuse abdominal tenderness and a mildly distended abdomen. Heart and lung sounds were clear to auscultation bilaterally; bowel sounds were auscultated in all four quadrants; the exam was negative for skin changes; and the patient was alert and oriented to person, time, place, and situation. Initial laboratory workup showed an elevated serum lipase of 8380 U/L (reference range: 23-200 U/L), transaminitis with aspartate aminotransferase (AST) of 281 U/L (reference range: 15-37 U/L) and alanine aminotransferase (ALT) of 163 U/L (reference range: 30-65 U/L), as well as elevated total bilirubin of 1.1 mg/dL (reference range: 0.2-1.0 mg/dL). All other lab values were stable, including his platelet count, which measured 311 K/mm^3^ (reference range: 150-400 K/mm^3^). The patient underwent a computed tomography (CT) scan of the abdomen, which revealed acute edematous interstitial pancreatitis without calcifications, as well as a dilated common bile duct measuring 1.4 cm, consistent with a reservoir effect from prior cholecystectomy, as shown in Figure 1. B12 and folate levels were within normal limits.

**Figure 1:**
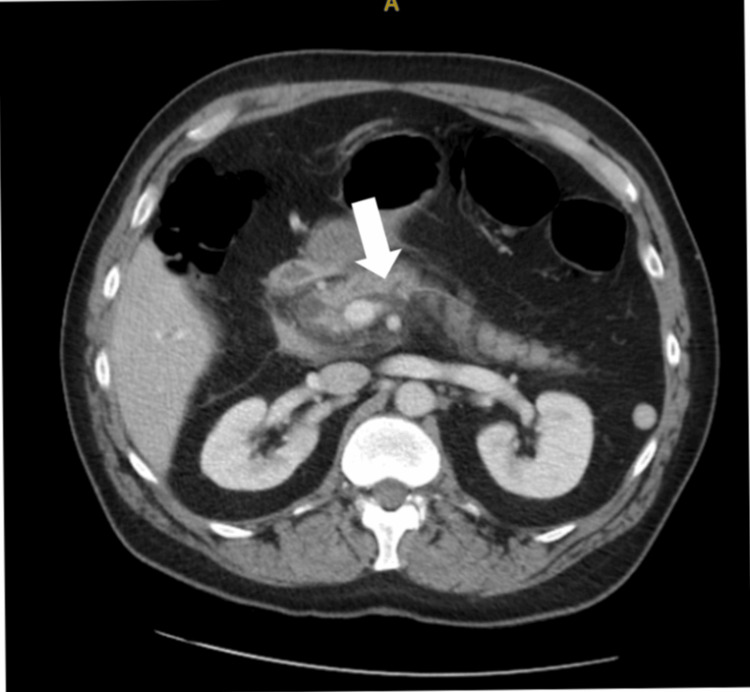
CT scan of the abdomen without contrast The white arrow highlights edematous interstitial pancreatitis. CT: computed tomography

The patient was admitted for acute pancreatitis secondary to alcohol abuse. General surgery was consulted for further evaluation. The team ultimately consulted gastroenterology, which recommended magnetic resonance cholangiopancreatography (MRCP), shown in Figure 2. The MRCP revealed no evidence of obstructive biliary process and no need for surgical intervention. Initial management included intravenous fluids, as well as pain and nausea control with morphine and ondansetron, promethazine, and a scopolamine patch, respectively.

**Figure 2:**
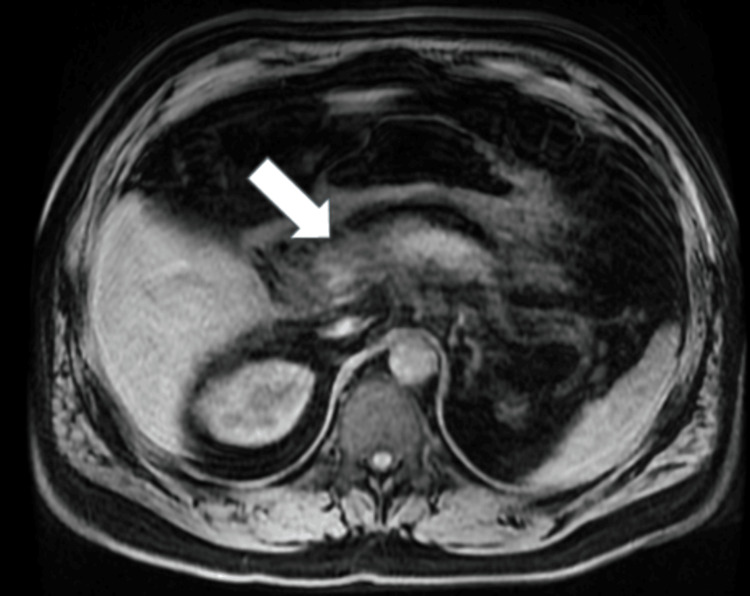
MRCP (axial view) The white arrow denotes the edematous pancreas; no biliary obstruction is seen. This is consistent with pancreatitis. MRCP: magnetic resonance cholangiopancreatography

Approximately 45 hours after admission, the patient became thrombocytopenic with a platelet count of 68 K/mm^3^ from 311 K/mm^3^ on admission. Hematology was consulted, and a diagnostic workup was performed. It was determined that the patient also exhibited hemolytic anemia as haptoglobin was less than 20 mg/dL (reference range: 40-200 mg/dL), lactate dehydrogenase (LDH) was 643 U/L (reference range: 140-280 U/L), direct bilirubin was 0.9 mg/dL (reference range: 0.0-0.03 mg/dL), and total bilirubin was 2.8 mg/dL. Peripheral smear showed normocytic anemia with thrombocytopenia. C3 levels were 171 mg/dL (reference range: 80-180 mg/dL). The Coombs test was negative. Further complement factor assays were deferred to the outpatient setting. ADAMTS-13 level was 96.9 U/dL (reference range: 60-130 U/dL), lowering suspicion for thrombotic thrombocytopenic purpura (TTP). Creatinine was 1.5 mg/dL (reference range: 0.7-1.3 mg/dL) on admission and 1.4 mg/dL at the time platelets dropped, which was elevated from the patient's baseline of 0.9 mg/dL and thus met criteria for an acute kidney injury (AKI). Blood urea nitrogen (BUN) levels acutely rose from 9 mg/dL to 16 mg/dL in the first 48 hours. Urinalysis obtained from the first 48 hours of admission showed moderate blood, bilirubin, urobilinogen, and +1 protein. Given the blood in the urine and the elevation of creatinine 150% from baseline, thrombocytopenia, and hemolytic anemia, the patient was suspected of having aHUS. 

During these first 48 hours of admission, the patient also developed acute hypoxic respiratory failure, systemic inflammatory response syndrome positive (leukocytosis, tachycardia, febrile) secondary to community-acquired pneumonia as seen on chest X-ray. He began a five-day course of ceftriaxone, a three-day course of azithromycin, and inhaled budesonide.

As the patient's pancreatitis improved with the supportive management discussed above, we saw his aHUS improve. By day 4, the patient’s platelets started to uptrend, and by day 7, they were within normal range. Renal function began to improve quickly. By hospital day 2, creatinine was 1.40 mg/dL and had almost returned to baseline by day 5. Hemolytic labs began to improve and return to normal by day 7. Relevant hospital lab values are shown in Table 1.

**Table 1:** Pertinent lab work during hospital course BUN: blood urea nitrogen

Parameter	Reference range	Admission	Hospital day 2	Hospital day 3	Hospital day 5	Hospital day 7	Hospital day 11 (discharge day)
Platelets	150-400 K/mm^3 ^	311	68	61	116	271	550
Total bilirubin	0.2-1.0 mg/dL	1.1	2.8	2.8	2.2	0.9	0.5
Hemoglobin	13-17 mg/dL	14.1	9.9	11.5	10.0	10.0	10.5
BUN	6-20 mg/dL	9	16	12	11	6	7
Creatinine	0.7-1.3 mg/dL	1.50	1.20	1.10	1.00	0.9	0.9
Leukocyte count	4.5-11 x10^9^/L	9.8	13.6	11.7	12.7	11.6	9.9

By hospital day 10, his respiratory symptoms had resolved after the antibiotics and supportive measures outlined above. He was discharged from the hospital on day 11.

## Discussion

aHUS is a diagnosis of exclusion. It is important to rule out other causes, such as TTP, typical HUS, and drug-induced complement-mediated anemia (CMA) by testing for ADAMTS-13 activity, Shiga toxin-producing bacteria, and performing a thorough medication history, as treatment will depend on the cause [7]. In this case, the patient presented with the classic triad of AKI, thrombocytopenia, and hemolytic anemia. aHUS was assumed when other causes of hemolytic anemia were negative [7]. Interestingly, this patient suffered a mild AKI. This is consistent with other reported cases in which aHUS was secondary to a trigger event [2,3].

Given the association of aHUS with complement dysregulation, workup should include assessment for low C3 and normal complement component 4 (C4). However, this pattern is observed in only about 30% of patients with aHUS and thus cannot be used to rule out aHUS [8]. Low factor H and low factor I levels may also be identified, though these findings are nonspecific for aHUS [8]. In our case, further complement testing was to be obtained in the outpatient setting, as we had low suspicion that the patient had primary aHUS.

According to a retrospective review conducted in 2021, the most common clinical profile of patients with aHUS triggered by acute pancreatitis was middle-aged men with a recent history of alcohol intake [4]. Episodes of aHUS secondary to gallstone-induced pancreatitis and idiopathic pancreatitis were significantly less common [4]. The etiology of aHUS triggered by alcohol-induced pancreatitis is not well understood. It is hypothesized that alcohol-induced pancreatitis may lead to the release of cytokines, such as interleukin (IL)-1 and tumor necrosis factor-α, or pancreatic exocrine protease-induced fibrinogen degeneration, causing endothelial damage [4,5]. In addition to this, it is thought that the release of proteases into the circulation modifies the circulating von Willebrand factor, favoring the aggregation of platelets [4]. However, more research is needed as the details of the underlying pathophysiologic mechanism are still unknown [4].

Most of the reported cases in the current literature show that treatment of aHUS, regardless of whether it is primary or secondary, involves plasma exchange therapy or eculizumab, a complement inhibitor [9-11]. However, in their analysis of secondary aHUS, Praga and Rodríguez de Córdoba emphasize that, in cases in which aHUS arises from an identifiable trigger, management should primarily focus on treating the underlying condition, which is consistent with the case we describe. They discuss that plasmapheresis and complement blockade therapies should be reserved for patients who do not respond to the treatment of the underlying condition, due to the fact that complement activation in secondary HUS is transient and that genetic abnormalities that would increase the risk of relapse are rarely ever found [2].

Though significantly less reported, there are cases of aHUS associated with acute pancreatitis, including alcohol-induced pancreatitis specifically, that have demonstrated clinical resolution with therapies focused on controlling pancreatic inflammation and systemic illness rather than complement inhibition [2,3]. For instance, in their review of the literature, Sandino-Pérez et al. reported a case of aHUS secondary to alcohol-induced pancreatitis that resolved with corticosteroid treatment [4]. Jaryal et al. also reported a case of diarrhea-associated aHUS, which was managed with optimal hydration and a three-day course of methylprednisolone followed by a tapering course of oral steroids over a period of four weeks. Within 24 hours of the first dose, platelet counts began to rise to a stable level [6]. Although the patient in our case did receive inhaled steroids, it is unclear if they played a significant role, given that they have relatively minimal systemic absorption. While the previously discussed cases have shown resolution following corticosteroid use, others improved with supportive measures alone. Kajiyama et al. cited a case of aHUS secondary to idiopathic pancreatitis that resolved with ulinastatin, gabexate mesylate, and meropenem alone [5]. Collectively, these findings support that secondary aHUS can be successfully treated by addressing the underlying trigger.

## Conclusions

aHUS is a rare TMA characterized by hemolytic anemia, thrombocytopenia, and acute renal failure. aHUS can be primary or secondary, meaning it is triggered by an event such as infection, surgery, pregnancy, or certain drugs. In extremely rare instances, aHUS has been reported to be triggered by acute pancreatitis. In practice, treatment for both primary and secondary aHUS now typically includes plasmapheresis or eculizumab. However, several reports suggest that in cases of secondary aHUS, treating the underlying cause can lead to resolution, as demonstrated in our case. Limitations of this case include a lack of genetic confirmation and limited generalizability. More research is needed to describe the pathophysiology of acute pancreatitis and aHUS.
